# Resource Management through Artificial Intelligence in Screening Programs—Key for the Successful Elimination of Hepatitis C

**DOI:** 10.3390/diagnostics12020346

**Published:** 2022-01-29

**Authors:** Anca Elena Butaru, Mădălin Mămuleanu, Costin Teodor Streba, Irina Paula Doica, Mihai Mircea Diculescu, Dan Ionuț Gheonea, Carmen Nicoleta Oancea

**Affiliations:** 1 Doctoral School, University of Medicine and Pharmacy of Craiova, 200349 Craiova, Romania; anca.butaru@yahoo.com (A.E.B.); doicairinapaula@gmail.com (I.P.D.); 2 Department of Automatic Control and Electronics, University of Craiova, 200585 Craiova, Romania; madalin.mamuleanu@edu.ucv.ro; 3 Oncometrics S.R.L., 200677 Craiova, Romania; 4 Department of Pulmonology, University of Medicine and Pharmacy of Craiova, 200349 Craiova, Romania; 5 Research Center of Gastroenterology and Hepatology, University of Medicine and Pharmacy of Craiova, 200638 Craiova, Romania; dan.gheonea@umfcv.ro; 6 Department of Gastroenterology, Carol Davila University of Medicine and Pharmacy, 050474 Bucharest, Romania; mircea.diculescu@umfcd.ro; 7 Department of Gastroenterology, University of Medicine and Pharmacy of Craiova, 200349 Craiova, Romania; 8 Department of Analytical Chemistry, University of Medicine and Pharmacy of Craiova, 200349 Craiova, Romania; carmen.oancea@umfcv.ro

**Keywords:** micro-elimination, hepatitis C virus, screening, artificial intelligence, prediction model

## Abstract

Background: The elimination of the Hepatitis C virus (HCV) will only be possible if rapid and efficient actions are taken. Artificial neural networks (ANNs) are computing systems based on the topology of the biological brain, containing connected artificial neurons that can be tasked with solving medical problems. Aim: We expanded the previously presented HCV micro-elimination project started in September 2020 that aimed to identify HCV infection through coordinated screening in asymptomatic populations and developed two ANN models able to identify at-risk subjects selected through a targeted questionnaire. Material and method: Our study included 14,042 screened participants from a southwestern region of Oltenia, Romania. Each participant completed a 12-item questionnaire along with anti-HCV antibody rapid testing. Hepatitis-C-positive subjects were linked to care and ultimately could receive antiviral treatment if they had detectable viremia. We built two ANNs, trained and tested on the dataset derived from the questionnaires and then used to identify patients in a similar, already existing dataset. Results: We found 114 HCV-positive patients (81 females), resulting in an overall prevalence of 0.81%. We identified sharing personal hygiene items, receiving blood transfusions, having dental work or surgery and re-using hypodermic needles as significant risk factors. When used on an existing dataset of 15,140 persons (119 HCV cases), the first ANN models correctly identified 97 (81.51%) HCV-positive subjects through 13,401 tests, while the second ANN model identified 81 (68.06%) patients through only 5192 tests. Conclusions: The use of ANNs in selecting screening candidates may improve resource allocation and prioritize cases more prone to severe disease.

## 1. Introduction

Hepatitis C virus (HCV) is a hepatotropic, enveloped RNA virus affiliated to the *Flaviviridae* family [1]. Its relatively recent discovery earned Harvey J. Alter, Charles M. Rice and Michael Houghton the Nobel Prize in 2020. Despite recent therapeutic advances, HCV remains a global public health concern [2]. According to the World Health Organization (WHO), 71 million persons are currently infected with HCV, 5.6 million of whom are in Europe [3,4]. A large percentage of these is unaware of their infection status [3]. Untreated, HCV promotes ample changes in the liver, from lipid accumulation to fibrogenesis and liver disfunction [5]. It is one of the leading causes of liver malignancy worldwide [6,7].

Direct-acting antivirals (DAAs) were introduced in 2013, this being a revolutionary point in the treatment of chronically HCV-infected patients [8,9], following several other antiviral regimens [8,9,10], as well as other treatment options [11]. These drug regimens have shown sustained virological response (SVR) in over 95% of the cases, once the infection was discovered [2,10]. These can help even patients with a wide range of comorbidities, as they are highly effective, well tolerated, safe and without substantial side effects [12]. This improvement in HCV therapy resulted in a worldwide action of 194 states to eliminate HCV infection globally.

The WHO plans to substantially reduce the prevalence and mortality by 2030 [3]; to reach this goal, the 2020 update recommends that all adults should be included in screening programs [12]. Screening proved to be an opportunity for treatment and cure after finding the infected patients before they become symptomatic or develop irreversible liver disease, cirrhosis or hepatocellular carcinoma (HCC) [13,14]. Limiting the transmission and thus avoiding debilitating surgery for HCV complications is cost-effective even at a low prevalence [15].

Another challenge for the elimination efforts is the impact of the current COVID-19 pandemic on the healthcare systems and patients. The negative influence of this pandemic translated in delayed detection and treatment initiation throughout both physical and psychological barriers, limited medical staff resources and transportation disfunctions [15,16]. As hospitals became burdened and medical personnel overloaded, most diagnostic and screening programs abruptly came to a worldwide stop, while the ones that went on were severely delayed and impaired [16].

The computer-aided medical management of diseases proved to be effective and cost-efficient [17]. The use of data science in determining at-risk populations leads to more targeted interventions and coherent screening efforts in selected populations. Implementing artificial intelligence in screening programs can reduce the strain on medical resources—selecting the patients that are more likely to be found positive leads to less consumables used, fewer doctors to be involved in fieldwork related to administering diagnostic tests, less exposure for patients and increased mental comfort for all involved parties [18]. To classify data according to different influencing factors and choosing an outcome, artificial neural networks (ANNs)—computing systems based on the topology of the biological brain, containing connected artificial neurons—were successfully employed in various fields, including medicine [19,20].

### Aim

We expanded the previously presented HCV micro-elimination project started in September 2020 that aimed to identify HCV infection through coordinated screening in asymptomatic populations and developed an evolved ANN model able to identify at-risk subjects selected through a targeted questionnaire.

## 2. Materials and Methods

### 2.1. Study Implementation

Data presented here resulted from a concomitant prospective investigation, following the same methodology described in the previously published two-arm, interventional, cross-sectional study [21], approaching a different population from the Southwest region of Oltenia, Romania—namely, the Gorj County. The study was conducted between February and July 2021, including all persons over 40 years of age who agreed in writing to be included, through either family physicians or medical offices of the OEC—Oltenia Energy Complex (a large public company active at multiple sites in the Gorj County, Romania, employing persons between 18 and 65 years). Family members of those contacted through the medical offices of the OEC were also included if they expressed interest in the study. In the case of people who tested positive, we also tested their families.

We were able to develop successful partnerships with both institutions in the Gorj County, as well as with general practitioners in the area. The Oltenia Energy Complex has over 12,000 employees, with 15 work-points, each with a medical office where tests could be performed in up to 3 shifts, according to the work schedule of the employees. We also had the support of 23 family doctors from the county whom performed the testing and administered the questionnaires to their patients.

The same multidisciplinary team from the University of Medicine and Pharmacy of Craiova was available to support antibody testing to detect the presence of anti-HCV antibodies and questionnaire delivery. We used the Anti-HCV TEST WB/S/P (INFO in vitro diagnostic test, Türklab Tibbi Malzemeler San. ve TIC. A.S., Izmir, Turkey) provided by the Association for the Promotion of Youth in Craiova (APT-C). The tests have a stated 100% sensitivity and specificity (https://www.turklab.com.tr/anti-hcv-test, accessed on 27 December 2021). All testing kits were stored on-site at room temperature. Using the previously described methodology [21], trained medical personnel from either GPs office or the OEC medical work-points took capillary blood samples by using the disposable sterile lancet included with each kit. The same medical personnel interpreted the test after 15 min: one test line next to the control line indicated a positive test.

The 12-item questionnaire (11 of which were designed to identify possible environmental-, health- and lifestyle-related risk factors) was administered to all participants and followed-up when the data were incomplete or missing, achieving a 100% completion rate.

All data from the recorded questionnaires, along with age, gender, type of provenance and anti-HCV antibody test result, were gathered in another online, secure database with the same schema, set up within the University, within the one previously described [21]. The team from our University handled data entry from the written questionnaires into the electronic database.

Once the presence of anti-HCV seropositivity was confirmed, the person was given the chance to perform, free of charge, HCV RNA viremia along with a basic set of biological tests: albumin, α-fetoprotein-AFP, alanine aminotransferase—ALT, aspartate aminotransferase—AST, hepatitis B surface antigen (HbsAg), anti-HIV antibodies and international normalized ratio (INR). Positive patients were referred to the Research Center of Gastroenterology and Hepatology within the University of Medicine and Pharmacy of Craiova for additional investigations such as Fibromax and abdominal ultrasound. Treatment was then promptly offered. All the investigations and treatment were conducted in accordance with national health system rules.

The project had an important social component, addressing a large population from a similarly disadvantaged region of Romania; it was thus promoted at all levels—local television, social media, public institutions of the mayor and local councils as well as through representatives of the civil society. A major accomplishment was conducting the study during the time of an on-going global medical situation, when the COVID-19 pandemic severely impaired the national medical system, as well as the economic sector.

The study received approval from the Ethics Committee within the University of Medicine and Pharmacy of Craiova (approval 82 dated 16 September 2020). Each tested person signed the GDPR approval form, and written informed consent was obtained in conformity with the principles of the 1975 Declaration of Helsinki. The managing structure of OEC gave written consent to conduct the study on their premises, and all medical doctors had written agreements with the University of Medicine and Pharmacy of Craiova.

### 2.2. Statistical Analysis

All statistical calculations were performed in GrapPad Prism (GraphPad Software, San Diego, CA, USA). We presented data as medians, with minimum and maximum values and average and standard deviations. We calculated Odds Ratio for different risk factors with 95% confidence intervals (95% CI). We used sensitivity, specificity, positive and negative predictive values as well as accuracy for the computer AI models that we developed. Other statistical parameters are detailed in the next section.

### 2.3. Computer-Aided Analysis and Development of an Artificial Intelligence (AI) Based Model

One of the primary endpoints of our research was to develop an effective AI model capable of selecting at-risk groups from large populations using the questionnaire data and basic demographic indicators, given the premise that large scale testing requires significant resources and analyzing the large amount of medical data would over-burden the medical staff.

The dataset used for training and evaluating the model was obtained from the electronic database corresponding to the current study. It contained 14 columns, as follows: “Result”, “Place of origin”, “Gender”, “Q2”, “Q3”, “Q4”, “Q5”, “Q6”, “Q7”, “Q8”, “Q9”, “Q10”, “Q11” and “Q12”. All values from the dataset were binary: 0 and 1. The patients’ provenance was encoded as 0 for rural and 1 for urban, while the gender was encoded as 1 for female and 0 for male. The other columns prefixed by “Q” were the answers to the 11 relevant items in the questionnaire. These were encoded as 0 for negative and 1 for positive answers. The column “Result” indicates the presence of anti-HCV antibodies, as obtained through the qualitative test described in the previous paragraph. Question 1 (“Q1”) was removed from the dataset as it contained known self-reported diagnosis with either hepatitis viruses or HIV, thus potentially influencing the behavior of the artificial neural network (ANN) model.

The model was then tested on the data obtained from the previous lot from the study we have already conducted, as the database structure was identical. For this, we selected 15,140 of the 15,383 individuals, for whom we made sure that the questionnaires were correctly completed. We repeated the questionnaire in 10 persons who initially provided invalid data, while having positive anti-HCV antibody tests, to maximize the number of true positive cases.

**Figure 1:**
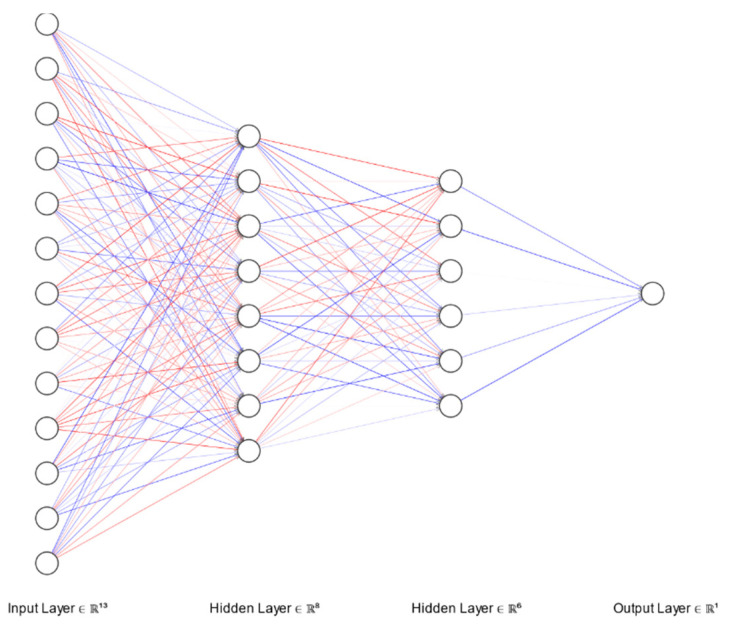
Proposed model for the ANN developed for the AI model.

**Figure 2:**
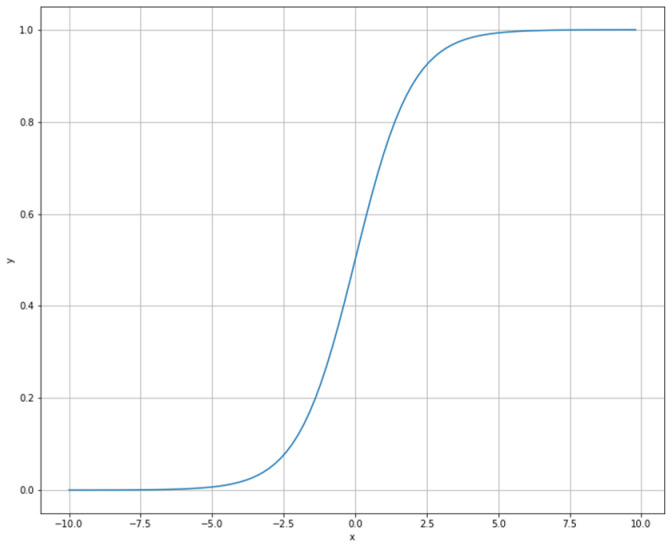
Sigmoid activation function of the ANN model.

Our approach to find a suitable neural network model was to start simple and measure the performance of the model after each major adjustment. After two iterations, in which we have analyzed how a model performs on imbalanced dataset, a proper model was found. The proposed model (Figure 1) has an input with 13 feature units, 2 hidden dense layers with 8 and 6 activation units, respectively, and 1 output layer. The activation function used for the hidden layers was a rectified linear unit while the output layer was sigmoid. For the model described earlier, we have worked with two hyperparameters configurations as described in Table 1. Since we have trained the same ANN architecture with two different hyperparameter configurations, two distinct models were obtained with different weights—Model 1 and Model 2. The graph of the sigmoid function is an S-shaped curve with the range from 0 to 1 (Figure 2). By using the sigmoid function as the activation function for the last layer (neuron), the output of our proposed models is a real number in the range of the sigmoid graph values, from 0 to 1. Hence, our models learned to predict the risk of hepatitis based on the answers given to the questionnaire.

**Figure 3:**
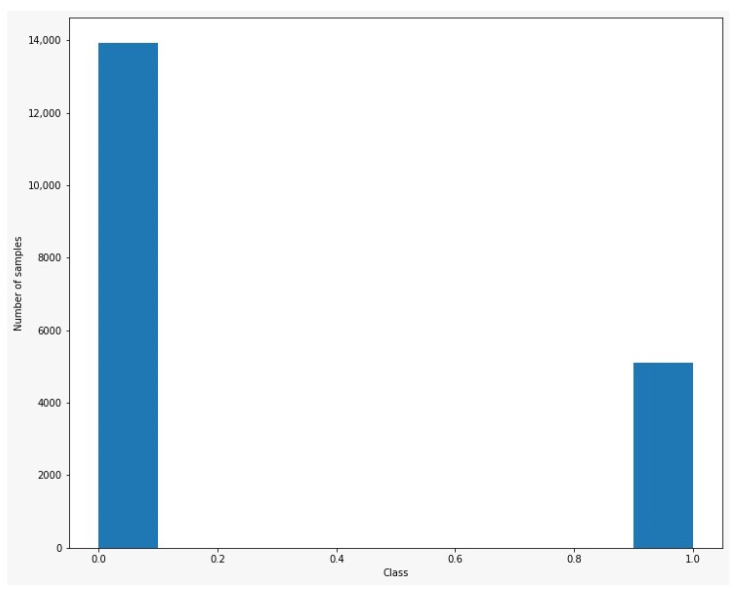
Dataset distribution after over-sampling.

The main goal of our model was to predict the risk of hepatitis, assessed as the presence of anti-HCV antibodies, based on the answers provided in the form. Typically, for a binary classification task, the loss function most commonly used is binary cross entropy (1):

(1) $$BCE=-\frac{1}{N}\;\overset{N}{\underset{i=1}{\sum }}y_{i}\cdot \log\left(p\left(y_{i}\right)\right)+\left(1-y_{i}\right)\cdot \log\;\left(1-p\left(y_{i}\right)\right)$$

where *N* is the total number of samples, *y*_i_ is the label for sample *i* and *p(y_i_)* is the predicted probability of sample *i* being *y*. However, the binary cross entropy loss penalizes equally for every classification error. This can lead to a model performing poorly on the minority class. For our model, we used a weighted binary cross entropy [4], in which the positive and negative predictions are multiplied by a coefficient (2):

(2) $$WBCE=-\frac{1}{N}\;\overset{N}{\underset{i=1}{\sum }}w_{0}(y_{i}\cdot \log\left(p\left(y_{i}\right)\right))+w_{1}\left(\left(1-y_{i}\right)\cdot \log\;\left(1-p\left(y_{i}\right)\right)\right)$$

where *w*_0_ is the coefficient for negative class and *w*_1_ is the coefficient for positive class. *w*_0_ and *w*_1_ are given by (3) and (4), respectively:

(3) $$w_{0}=\frac{1}{2}$$

(4) $$w_{1}=\frac{N}{z\;\cdot 2}$$

where *N* is the total number of samples, and *z* is the number of positive samples in the dataset:

The dataset was randomly split into 70% for training and 30% for testing. In addition, we have added 5000 duplicate samples from the minority class in the dataset. We have chosen this ratio and this procedure to be sure that samples from the minority class will be found in both subsets (training and testing). This resulted in 3627 positive samples in training subset and 1487 samples in the test subset. The models were trained for 30 epochs with batch sizes of 50 and 1024. The optimizer used was Root Mean Squared prop with a learning rate of 0.001. A large batch size was chosen for one of the configurations to ensure that we have enough samples from the positive class. The dataset distribution per class is shown in Figure 3.

### 2.4. Performance Metrics

For measuring the performance of a deep learning model, accuracy is the most used metric. Accuracy measures the ratio of the correctly predicted outputs and all predictions (5). While this metric alone can be useful to measure the performance of a model, for models trained on imbalanced data, accuracy is not capable of correctly assessing the model performance. Considering our dataset with 19,042 samples (after over-sampling), the ratio of negative and positive samples was ~1:2.7. This means that for each positive sample, there were 2.7 negative samples. Hence, for our model, in addition to accuracy, we measured precision (6), recall (7) and area under curve.

The fully connected ANN models described earlier were implemented in Keras 2.7.0 with Tensorflow (Google Brain Team, Google, Chicago, IL, USA) using Python version 3.7.12 (Python Software Foundation, USA). The model was trained in cloud using Google Colab (https://colab.research.google.com/ (accessed on 18 November 2021), Google, Chicago, IL, USA):

(5) $$Accuracy=\frac{TP+TN}{TP+FN+FP+TN}$$

(6) $$Precision=\frac{TP}{TP+FP}$$

(7) $$Recall=\frac{TP}{TP+FN}$$

where *TP*—true positives; *TN*—true negatives; *FN*—false negatives; *FP*—false positives.

## 3. Results

### 3.1. Study Lot

**Figure 4:**
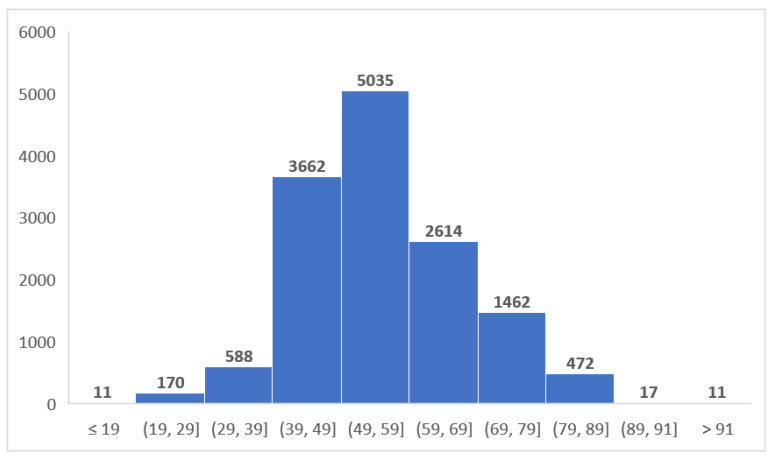
Overview of the age distribution in the study lot.

We included 14,042 subjects, aged between 18 and 96 years (mean age 55.37 ± 11.97 years); of these, 7622 (54.28%) were men. An overview of the age data can be found in Table 2 and Figure 4.

We found 114 persons with anti-HCV antibodies present (resulting in a prevalence of 0.81%), aged between 41 and 85 years. Of these, 81 were female (1.26% prevalence among females) and 33 men (0.43% prevalence in men). The overall incidence was 377.44 cases/100,000 persons; in women, we found an incidence of 482.9 cases/100,000 compared to only 288.64 cases/100,000 in men. The demographic data are summarized in Table 3.

**Figure 5:**
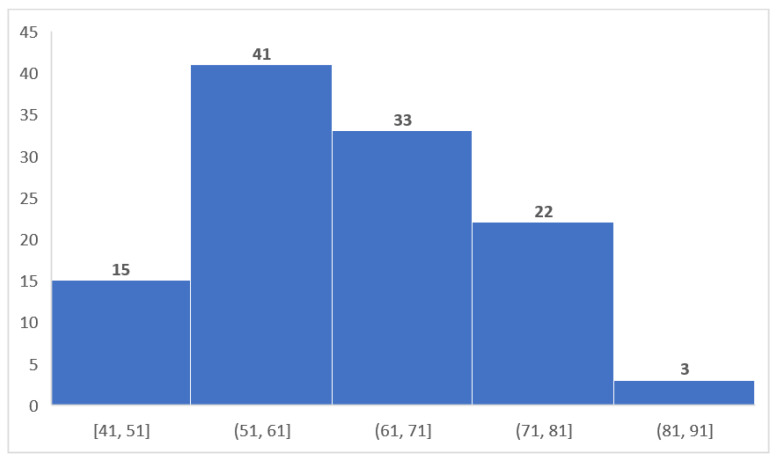
Age distribution in persons with positive anti-HCV antibodies.

We could observe that 64.9% of cases were between 51 and 71 years of age; most subjects (41 cases, 35.96% of all HCV-positive subjects) were between 51 and 61 years, followed by the 61–71 decade (33 cases, 28.94%). The age distribution of positive anti-HCV antibodies cases is visualized in Figure 5.

**Figure 6:**
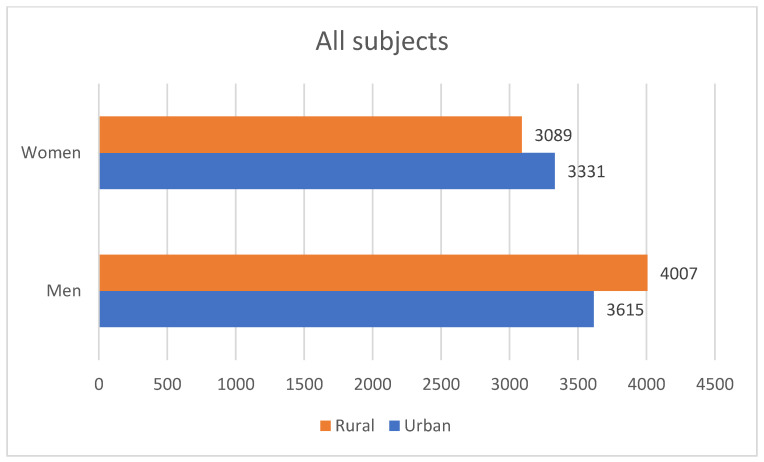
Distribution by gender and provenance in the entire lot.

**Figure 7:**
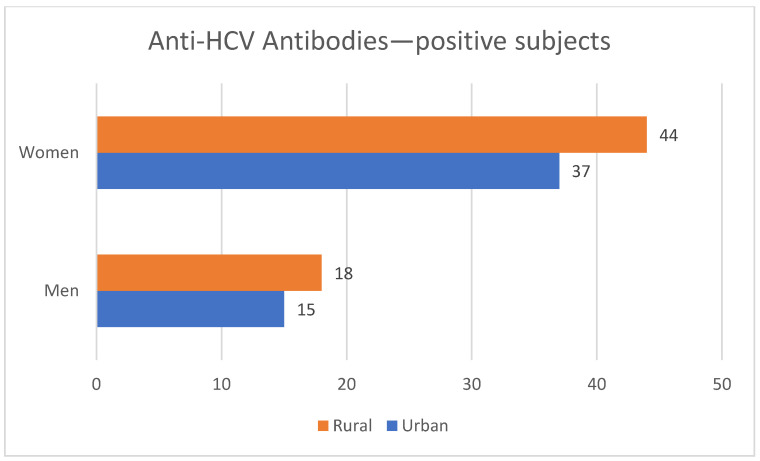
Gender and residence of men and women in the subgroup of subjects with present anti-HCV antibodies.

Regarding the provenance of the subjects, we had roughly symmetrical distribution—7096 (50.53%) coming from rural areas (of which men predominated—4007 persons). The remaining 6946 persons (3615 male and 3331 women) were from urban areas. More women with positive anti-HCV antibodies were from the rural areas (44 of the 62 subjects); a rural background predominated in men, as well (18 men from rural areas versus 15 from urban establishments). A synthesis of these data can be found in Figure 6 and Figure 7.

We identified through the questionnaire several possible factors that related to a higher probability of finding anti-HCV antibodies. Respondents could give binary answers to 11 out of the total 12 questions; the first question asked them to mention any prior hepatitis or HIV infection and was thus excluded from this analysis. We present in Table 4 the percentage of positive answers to questions 2 to 12 in the whole screened population as opposed to the positive anti-HCV antibodies lot. The entirety of the data can be seen in Table 5.

Following with an odds ratio analysis, we found that sharing personal hygiene items (Q3), receiving blood transfusions (Q5), having had dental work or surgery (Q6) and using hypodermic needles already used by other persons (Q11) were statistically significant risk factors. An overview of the analysis can be found in Table 6.

**Figure 8:**
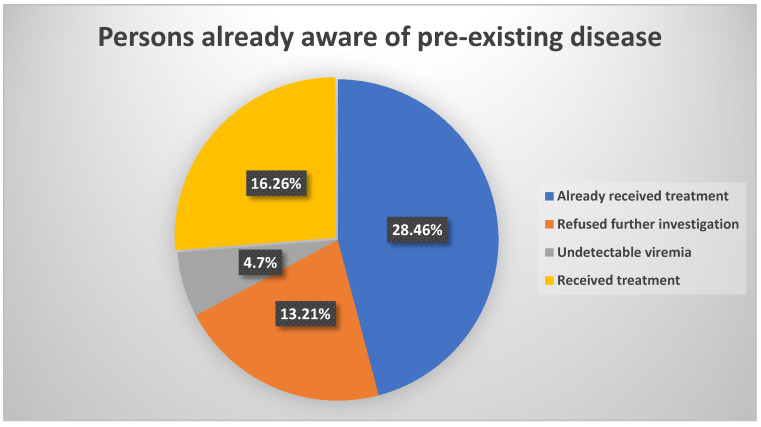
Breakdown of the sub-lot containing subjects already aware of their HCV-positive status. Of the 61 persons, 16 chose to start an antiviral regimen, having detectable viremia and not receiving previous treatment.

**Figure 9:**
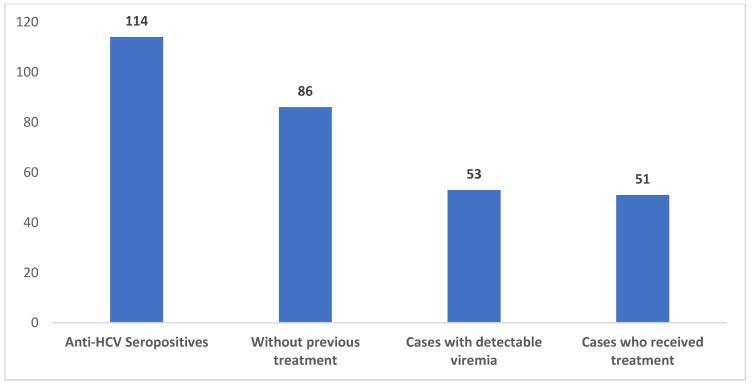
Linkage-to-care analysis of the current lot, compared to the previously analyzed lot.

We performed a linkage-to-care analysis of the current population, comparing it to the existing data from the other concomitant study. In the current lot, we found 114 patients with anti-HCV antibodies, of which 61 already knew of their condition, of whom 28 already received treatment, 13 refused further investigation and 4 had undetectable viremia. The remaining 16 chose to begin antiviral treatment. An overview of this sub-lot can be seen in Figure 8. Overall, we identified 53 persons who had detectable viremia and did not receive any previous treatment; of these, 51 chose to receive antiviral treatment. Linkage data are presented in Figure 9.

### 3.2. Performance of the ANN Model

#### 3.2.1. Training and Testing Performance

**Figure 10:**
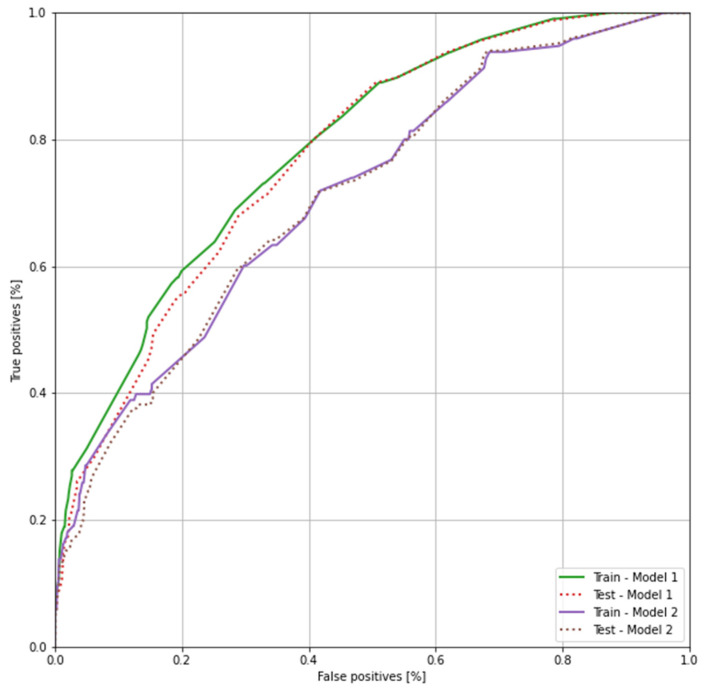
Receiver operating characteristic curve of both proposed models.

As mentioned earlier, accuracy is not enough to assess the performance of our models, so we have plotted have plotted the receiver operating characteristic curve for both models (Figure 10) for the training and testing stage. The receiver operating characteristic curve (ROC) is a graph of true positive rate against false positive rate and, in AI, is mainly used for assessing the performance of binary classifiers. The closer the curve gets to the top left corner, the better the model is performing. The area under the ROC (AUC) is presented in Table 7. The closer the AUC of a model gets to one, the better it performs. At training phase, we can see that both models are starting with a value for AUC between 0.40 and 0.55, and the values are increasing after each training epoch.

Analyzing the ROC, we could conclude that, for the current lot of 14,042 subjects, Model 1 performed better than Model 2. However, as we could see further in our analysis, when choosing a threshold for classifying if the subject is at risk of hepatitis, the first model was more biased than the second model, and it required more tests to properly asses the subjects.

The values for precision, accuracy and recall at epoch 30 in the training phase are presented in Table 8.

#### 3.2.2. Testing Results of the Two Models on the Other Dataset

Given the similar structure of the two analyzed populations, we trained our developed models on the current lot of 14,042 subjects (114 anti-HCV antibody positive persons) and tested both on the 15,140 subjects (119 HCV cases) from the previous study. The 2 ANNs gave probability answers between 0.00 and 1.00 on the presence of anti-HCV antibodies; the lower the confidence (closer to 0), the less likely that person was to be HCV positive, thus, with less need to be tested.

We chose 0.05 increments between 0.65 and 0.95 as reference confidence levels to assess sensitivity, specificity, positive (PPV) and negative (NPV) predictive values, as well as requested tests for positives. An overview of the data can be seen in Table 9.

At 0.65, 0.7, 0.75, 0.8 and 0.85 confidence cut-offs, Model 1 correctly identified 97 HCV-positives and considered between 13,401 and 13,310 others as possible positives (between 1642 and 1830 negatives, of which 22 were false negatives). Given that the disease prevalence was 0.79%, the best accuracy achieved by Model 1 was 30.88%, at 0.95 confidence level, while still requiring 10,527 tests to be performed; at this value, it correctly identified 90 anti-HCV antibody positive persons.

Comparatively, Model 2 performed a more balanced analysis of the data. Hence, at the 0.65 reference confidence value, it correctly identified 81 HCV-positives, only requiring 5192 tests to be performed (correctly identifying 9948 negative persons). The number of correctly identified anti-HCV positives dropped as the confidence grew, however, consistently less tests were required to be performed (4 positives identified through 55 requested tests at 0.95 confidence level). Given the 0.79% disease prevalence in the test lot, the best accuracy of Model 2 was 98.9% at 0.95 confidence level.

**Figure 11:**
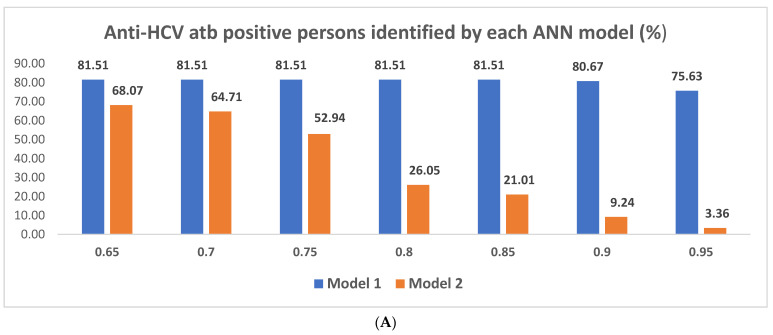

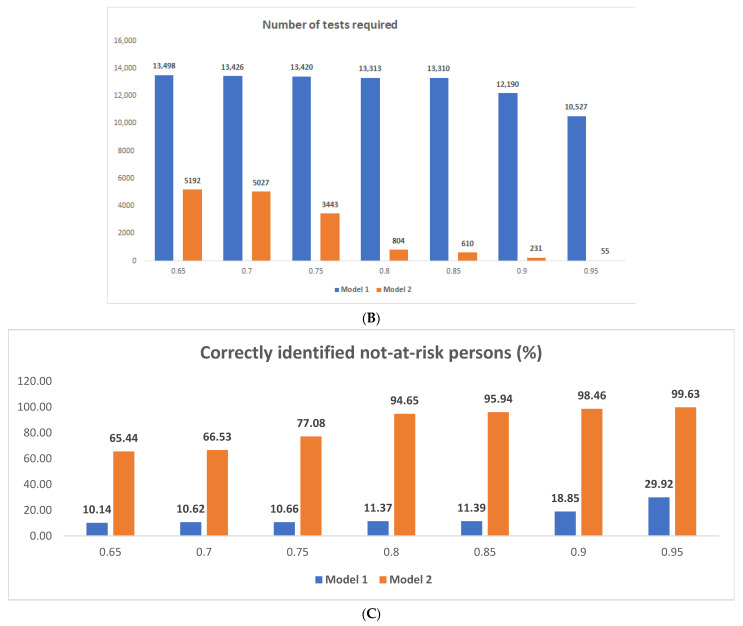
(**A**) Comparative overview of the number of correctly identified HCV-positive persons by each ANN model; we can observe the constantly high number of true positives identified by Model 1, however (**B**), at the cost of a significantly higher number of tests performed compared to Model 2. In contrast (**C**), Model 2 was more effective at identifying true not-at-risk persons; this feature, corroborated with the low HCV incidence of 0.79% in the chosen dataset of 15,140 subjects, resulted in an overall higher accuracy for Model 2.

A comparative overview of the data can be seen in Figure 11A–C.

## 4. Discussion

To identify the persons that need immediate access to treatment, micro-elimination strategies aim to test specifically defined populations from an established geographical area. It appears that the micro-elimination design is more suitable than the macro-elimination one [1,2,4]. The ongoing diagnostic algorithm, using rapid diagnostic serological tests to detect HCV antibodies, can help to find the person exposed to the hepatitis C virus. After establishing seropositivity, HCV RNA is useful for discovering actively infected patients [1,2,3,4,22].

Micro-elimination strategies would involve all stakeholders such as local administrative, private sector or civil society representatives that should conduct the screening program. Primary care professionals also play an important role in community education, and it has been shown to be effective to cope with the stigma between patients. Numerous challenges such as social labeling, lack of awareness of untreated HCV infection and fear of diagnosis and treatment represent an increased risk [23]. These barriers can be overcome through intensive educational and awareness activities.

With a global incidence rate of 23.7 per 100,000 and 71 million persons living with HCV worldwide, this infection remains a substantial healthcare problem [3]. Left untreated, hepatitis C infection can cause life-long and serious complications such as hepatocellular carcinoma [3].

The availability of direct-acting antiviral (DAA) therapy greatly changed the perspective of HCV curability and elimination [23,24,25,26]. Easily understandable, once-daily and with a high viral response rate, this therapy has transformed the WHO’s goal into an optimistic landscape and has become the state-of-art treatment. Even so, extensive screening and therapy access is required. Early detection of the virus is important for the physical and mental health of the individuals because only the screened, diagnosed and linked to care can benefit from DAAs’ potential [25]. Without screening, a late diagnosis is translated in hospitalization and death due to rising rates of hepatocellular carcinoma [27].

The dataset contained a total of 14,042 rows representing a unique response sequence to the form pertinent to each patient who was enrolled. From this batch of results, 114 were positive. Operating with datasets that have imbalanced classes is not a new issue in artificial intelligence research, especially in healthcare. In deep learning research, it is a general assumption that the dataset which will be used contains classes with equal or almost equal numbers of samples. However, in the field of healthcare, this is not always the case. Class imbalance can occur in many types of datasets, especially when dealing with rare diseases or with few cases of a specific condition in an extremely large population, resulting in low prevalence. Training a binary classifier using an imbalanced dataset (114 positive cases from 14,042 total in our study) often leads to poor prediction results on the test batch and underfitting in the minority class. To address this issue of imbalanced datasets, many methods were proposed, such as direct methods [28], random oversampling [29] or random undersampling [29]. Random oversampling adds random copies of existing minority data to the same class in the existing base. Similarly, random undersampling removes random copies of existing majority data [29,30]. These methods are classified in two main categories: methods which are applied at the algorithm level and methods applied at the data level. Der-Chiang Li et al. [5] proposed a procedure in which the majority sample group was undersampled and the minority sample group was oversampled and obtained a significant increase in accuracy using a support vector machine model (SVM). Chuanxia Jian et al. [31] proposed a different contribution sampling method (DCS) for binary classification. The DCS sampling scheme contains a group of SVMs. They tested their method on 19 healthcare datasets (10 datasets with 2 classes and 9 datasets with more than 2 classes) and observed that DCS obtained the largest average recall from the methods tested [31]. The simplest method to apply the over-sampling technique is to duplicate the existing samples from the minority class [32]. However, depending on the multiplying factor, this approach of oversampling can pose a problem in the trained model [32,33]. When using the random oversampling technique, there is a risk of overfitting the training data since samples from the minority class are duplicated [34]. Weiss et al. [35] used C4.5, an algorithm which generates decision trees [36], and observed that different distributions of classes perform better in different areas of ROC but not in all. However, the difference between AUC measured for 50% minority class and 90% minority class was small (0.862 vs. 0.855) [35]. The Synthetic Minority Oversampling Technique (SMOTE), proposed by Chawla et al. [30], introduced a minority oversampling approach where “synthetic” samples were added to the minority class instead of duplicating the existing ones.

Our study had several limitations. The study lot, being extensive, most probably included several superficially completed questionnaires, as well as others that had significant omissions. We eliminated incomplete or obviously wrongly completed forms (i.e., answering “yes” to all questions) to minimize the bias that would have otherwise significantly decreased the accuracy of our ANN models. We are confident that even those who did not declare significant risk factors through the questionnaires were most likely exposed to them at some point in their lives and either do not recall or chose not to disclose them. A second issue, which could be deriving from the previous point, is balancing the decision-making process—a goal of any screening program would be to correctly identify as many positives as possible. Both models did leave out a proportion of positive anti-HCV antibody subjects; even so, the aim of our attempt was to find a suitable computer model to prioritize patient testing, minimizing resources and allotting time for the medical personnel, particularly useful in crisis situations such as an ongoing pandemic affecting low-income communities. For this, we proposed and tested two models—one that did not try to minimize the number of tests used, thus discovering more patients, while the other, more balanced model greatly decreased the number of tests to be used while maintaining a high probability of discovery.

## 5. Conclusions

We have described here two different ANN models designed to identify persons at-risk of developing HCV based on data from an easy to administer questionnaire. At the minimal confidence level, the first model identified 13.5% more cases, at the cost 61.5% more tests required. Thus, the first ANN model can be used when more resources can be allotted to testing, while the second can successfully prioritize at-risk populations when testing resources are limited.

## Figures and Tables

**Table 1:** Hyperparameters configurations of the two ANN models.

Hyperparameter	Model 1	Model 2
Batch size	50	1024
Epochs	30	30
Class weights	*w*_0_ = 1	*w*_0_ = Equation (3)
	*w*_1_ = 120	*w*_1_ = Equation (4)
Learning rate	0.001	0.001

**Table 2:** Demographic data of the entire study lot.

Whole Lot	Male	Female	Total
Number (%)	7622 (54.28)	6420 (45.72)	14,042
Median age (yrs.)	53	55	53
Minimum age (yrs.)	10	17	10
Maximum age (yrs.)	96	97	97
Mean age (yrs.)	54.52	56.39	55.37
Standard Dev. (yrs.)	11.54	12.39	11.97

**Table 3:** An overview of the positive anti-HCV antibodies subjects in our study lot.

Anti-HCV Antibodies Positive	Male	Female	Total
Number (% of positives)	33 (28.95)	81 (71.05)	114 (100)
Prevalence (%)	0.43	1.26	0.81
Incidence (per 100,000)	288.64	482.9	377.44
Median age (yrs.)	62	61.5	62
Minimum age (yrs.)	41	43	41
Maximum age (yrs.)	85	82	85
Mean age (yrs.)	59.12	63.23	62.04
Standard Dev. (yrs.)	10.21	10.11	10.13

**Table 4:** Comparative view of the percent of positive answers to questions 2 to 12 in the whole lot as opposed to the positive anti-HCV antibody lot.

	Lot (%)	HCV (%)
Q2. Do you share residence with a HCV infected person?	1.00	1.75
Q3. Have you shared with anyone personal hygiene items that may come in direct contact with the blood (toothbrush, razor, scissors, manicure kits)?	5.02	19.30
Q4. Do you work in environments with risk of blood contamination (doctor, nurse)?	2.05	0.00
Q5. Have you ever received blood transfusions, blood products or an organ transplant?	5.93	17.54
Q6. Have you ever had dental work, including dental surgery?	54.97	71.05
Q7. Have you ever undergone surgery?	48.75	54.39
Q8. Are you included in a dialysis program?	0.23	0.00
Q9. Have you ever done tattoos or piercings?	5.97	2.63
Q10. Have you ever had unprotected sex with an unknown partner, with more than one partner, or have you ever been diagnosed with a sexually transmitted disease?	4.87	0.88
Q11. Have you ever used injection needles or syringes already used by others?	0.04	2.63
Q12. Have you ever lived in correctional institutions, or have you ever been in detention?	0.48	0.00

**Table 5:** Overview of the positive answers in the two lots, divided by gender. Percentages are of the respective lots.

	Screened Population			Anti-HCV Antibodies Positives		
	Men	Women	Total n, (%)	Men	Women	Total n, (%)
Q2	70	71	141 (1.00)	2	0	2 (1.75)
Q3	305	400	705 (5.02)	19	3	22 (19.3)
Q4	59	229	288 (2.05)	0	0	0 (0.00)
Q5	345	488	833 (5.93)	19	1	20 (17.54)
Q6	3848	3871	7719 (54.97)	55	26	81 (71.05)
Q7	3202	3644	6846 (48.75)	48	14	62 (54.39)
Q8	13	19	32 (0.23)	0	0	0 (0.00)
Q9	681	158	839 (5.97)	0	3	3 (2.63)
Q10	547	137	684 (4.87)	1	0	1 (0.88)
Q11	2	4	6 (0.04)	3	0	3 (2.63)
Q12	52	15	67 (0.48)	0	0	0 (0.00)

**Table 6:** Odds Ratio overview for the relevant risk factors identified in the screened population.

	Q3	Q5	Q6	Q11
Odds ratio	4.63	3.43	2.02	125.45
95% Confidence Interval	2.89 to 7.43	2.10 to 5.59	1.34 to 3.03	25.04 to 628.35
Significance level	*p* < 0.0001	*p* < 0.0001	*p* = 0.0007	*p* < 0.0001

**Table 7:** AUC for the two proposed models.

	Model 1		Model 2	
	AUC	Validation AUC	AUC	Validation AUC
Epoch 1	0.55	0.67	0.49	0.40
Epoch 2	0.71	0.72	0.41	0.43
Epoch 3	0.73	0.73	0.43	0.44
Epoch 4	0.73	0.74	0.47	0.47
Epoch 5	0.74	0.74	0.48	0.48
Epoch 6	0.74	0.75	0.48	0.46
Epoch 7	0.75	0.75	0.48	0.49
Epoch 8	0.75	0.75	0.51	0.52
Epoch 9	0.76	0.76	0.54	0.56
Epoch 10	0.76	0.76	0.57	0.58
Epoch 11	0.76	0.76	0.60	0.60
Epoch 12	0.77	0.76	0.62	0.63
Epoch 13	0.77	0.76	0.64	0.64
Epoch 14	0.77	0.76	0.65	0.65
Epoch 15	0.77	0.77	0.66	0.65
Epoch 16	0.77	0.77	0.66	0.66
Epoch 17	0.77	0.77	0.67	0.67
Epoch 18	0.78	0.77	0.67	0.67
Epoch 19	0.78	0.77	0.68	0.68
Epoch 20	0.78	0.77	0.68	0.68
Epoch 21	0.78	0.77	0.69	0.69
Epoch 22	0.78	0.77	0.69	0.69
Epoch 23	0.78	0.77	0.70	0.70
Epoch 24	0.78	0.77	0.70	0.71
Epoch 25	0.78	0.77	0.71	0.71
Epoch 26	0.78	0.78	0.71	0.71
Epoch 27	0.78	0.77	0.71	0.71
Epoch 28	0.78	0.77	0.71	0.71
Epoch 29	0.78	0.78	0.71	0.71
Epoch 30	0.78	0.78	0.71	0.71

**Table 8:** Values for accuracy, precision and recall at epoch 30 in the training phase.

Metric	Model 1	Model 2
Accuracy	0.3525	0.2961
Precision	0.2959	0.2788
Recall	1.0000	1.0000

**Table 9:** Sensitivity, specificity, PPV, NPV, accuracy and the number of tests required to identify the true positives for the two ANN models at different confidence levels.

Confidence Cut-Off Value	ANN Model	Sensitivity (%)	Specificity (%)	PPV (%)	NPV (%)	Accuracy (%)	Required Tests
0.65	Model 1	81.51	10.78	0.72	98.66	11.34	13,498
	Model 2	68.07	65.97	1.56	99.62	65.99	5192
0.70	Model 1	81.51	11.26	0.72	98.72	11.82	13,426
	Model 2	64.71	67.05	1.53	99.58	67.03	5027
0.75	Model 1	81.51	11.3	0.72	98.72	11.86	13,420
	Model 2	52.94	77.5	1.83	99.52	77.31	3443
0.80	Model 1	81.51	12.02	0.73	98.8	12.56	13,313
	Model 2	26.05	94.85	3.86	99.39	94.31	804
0.85	Model 1	81.51	12.04	0.73	98.8	12.58	13,310
	Model 2	21.01	96.11	4.1	99.35	95.52	610
0.90	Model 1	80.67	19.49	0.79	99.22	19.97	12,190
	Model 2	9.24	98.54	4.76	99.28	97.83	231
0.95	Model 1	75.63	30.52	0.85	99.37	30.88	10,527
	Model 2	3.36	99.66	7.27	99.24	98.9	55

## Data Availability

Anonymized data pertaining to the study can be obtained from the authors upon request.
